# Genetic Effects of the Schizophrenia-Related Gene *DTNBP1* in Temporal Lobe Epilepsy

**DOI:** 10.3389/fgene.2021.553974

**Published:** 2021-02-19

**Authors:** Hua Tao, Xu Zhou, Jun Chen, Haihong Zhou, Lidan Huang, Yujie Cai, Jiawu Fu, Zhou Liu, Yanyan Chen, Chaowen Sun, Bin Zhao, Wangtao Zhong, Keshen Li

**Affiliations:** ^1^Department of Neurology, Affiliated Hospital of Guangdong Medical University, Zhanjiang, China; ^2^Guangdong Key Laboratory of Age-related Cardiac and Cerebral Diseases, Guangdong Medical University, Zhanjiang, China; ^3^Institute of Neurology, Guangdong Medical University, Zhanjiang, China; ^4^Neurology and Neurosurgery Division, Stroke Center, The First Affiliated Hospital, Clinical Medicine Research Institute, Jinan University, Guangzhou, China

**Keywords:** *DTNBP1*, schizophrenia, genetic mutation, temporal lobe epilepsy, polymorphism

## Abstract

Recent studies have reported patients who concurrently exhibit conditions of epilepsy and schizophrenia, indicating certain shared pathologies between them. This study aimed to investigate the genetic effects of the schizophrenia-related gene *DTNBP1* in temporal lobe epilepsy (TLE). A total of 496 TLE patients and 528 healthy individuals were successfully genotyped for six *DTNBP1* polymorphisms (rs760665, rs1011313, rs2619528, rs2619522, rs909706, and rs2619538), including 335 TLE patients and 325 healthy controls in cohort 1, and 161 TLE patients and 203 healthy controls in cohort 2. The frequency of the TT genotype at rs909706 T > C was lower in TLE patients than in normal controls in the initial cohort (cohort 1), which was confirmed in an independent cohort (cohort 2). However, the intronic T allele failed to be in linkage disequilibrium (LD) with any functional variations nearby; thus, together with the CCAC and TCAT haplotypes (rs1011313-rs2619528-rs2619522-rs909706) observed in the study, this allele acts only as a protective factor against susceptibility to TLE. Meanwhile, a *novo* mutant allele rs2619538 T > A was exclusively observed in TLE patients, and a dual-luciferase assay revealed that the mutant allele was increased by approximately 22% in the *DTNBP2* promoter compared with the wild-type allele. Together with the trend of increasing *DTNBP1* expression in epilepsy patients and animal models in this study, these are the first findings to demonstrate the genetic association of *DTNBP1* with TLE. Homozygous mutation of rs2619538 T > A likely promotes *DTNBP1* expression and facilitates subsequent processes in epilepsy pathologies. Thus, the role of *DTNBP1* in TLE deserves further exploration in the future.

## Introduction

Epilepsy is a brain disorder characterized by recurrent seizures. Many gene mutations have been identified via exon sequencing as being responsible for epileptic seizures; these genes involve many ion and non-ion channels, such as *KCNA2, KCNT1, GRIN2A*, and *CHD2* (He et al., 2019). Nevertheless, epilepsy pathologies are difficult to fully identify, making the development of efficient therapies challenging. Thus, almost one-third of patients, especially those with temporal lobe epilepsy (TLE), a main drug-resistant epilepsy, still suffer from recurrent seizures because of resistance to available treatments. Hence, more efforts should be made to explore epilepsy pathologies, and disclosing epileptic seizures in atypical conditions might be a novel strategy.

In clinical practice, typical symptoms of a disease are often seen as complications due to other diseases, underscoring certain shared mechanisms between them. Considering this phenomenon, some previous studies have explored the manifestation of epileptic seizures in Alzheimer’s disease and found that insufficient expression of *ADAM10* is a driving force in epilepsy pathologies (Clement et al., 2008; Prox et al., 2013). The prevalence of epileptic seizures in psychosis has also attracted increased attention in recent years. According to a retrospective cohort study based on the Oxford Record Linkage Study and English national linked Hospital Episode Statistics, patients admitted to the hospital with schizophrenia have an elevated risk of epileptic seizures, and likewise, patients with epilepsy have an elevated risk of schizophrenia (Wotton and Goldacre, 2012). In addition, male adolescents with severe, refractory epilepsy have a high risk of schizophrenia (Andersen et al., 2019). Obviously, this evidence supports a bidirectional relationship and some shared mechanisms between epilepsy and schizophrenia.

Expanding experimental evidence supports the notion of shared mechanisms between epilepsy and schizophrenia. For example, Michelson et al. reported that familial partial trisomy 15q11-13 presented either as intractable epilepsy or as psychiatric illness (Michelson et al., 2011), and Stewart et al. observed that the frequency of copy number abnormalities and maternal duplication 15q11-q13 in patients with combined schizophrenia and epilepsy is significantly higher than in patients with schizophrenia (Stewart et al., 2011). Furthermore, schizophrenia- and epilepsy-related alterations were simultaneously observed in a mouse model with a microdeletion within 15q13.3 (Fejgin et al., 2014). Other studies revealed that abnormal expression of CYFIP1, VRK2, and metabotropic glutamate 2/3 (mGlu2/3) receptor is involved in both schizophrenia and epilepsy (Nebel et al., 2016; Azimi et al., 2018).

The *DTNBP1* gene encodes dystrobrevin binding protein 1, which participates in organelle biogenesis, and recent evidence indicates that it plays key roles in brain development, neuronal excitability, and schizophrenia-related pathologies (Chen et al., 2008; Saggu et al., 2013; Wang et al., 2017; Konopaske et al., 2018; Mohamed et al., 2018), i.e., decreased expression of *DTNBP1* was observed to reduce exocytosis of brain-derived neurotrophic factor (BDNF) from cortical excitatory neurons. The reduction in BDNF exocytosis resulted in a decreasing number of inhibitory synapses located on excitatory neurons, while the application of exogenous BDNF rescued these inhibitory synaptic deficits (Yuan et al., 2016). Interestingly, the hippocampus is an enriched region for *DTNBP1* under normal conditions, and postmortem studies have shown a decreased level of *DTNBP1* expression in the hippocampus of patients with schizophrenia (Wang et al., 2017), indicating that decreased *DTNBP1* is a pathological condition in schizophrenia. Since the hippocampus is also crucial in TLE pathologies characterized by deregulation of neuronal excitability, it is likely that *DTNBP1* plays a key role in both TLE and schizophrenia.

Considering that single-nucleotide polymorphisms are among the most important genetic variations that regulate gene expression and subsequent biological activity, this study selected candidate variations within the *DTNBP1* gene region on the basis of biological plausibility in previous research, including rs2619522 involved in hippocampal gray matter volume (Trost et al., 2013) and rs2619538 in volumes of both gray and white matter (Tognin et al., 2011) in healthy subjects, rs760665 and rs909706 involved in the hippocampal glutamate concentration of healthy individuals (Wirth et al., 2012), and rs1011313 involved in volume reduction of multiple brain regions (Thirunavukkarasu et al., 2014) and rs2619528 in prefrontal function (Fallgatter et al., 2010) of schizophrenia patients. We aimed to clarify the genetic effects of *DTNBP1* in TLE based on these six SNPs.

## Materials and Methods

### Human Subject Enrollment

All experimental protocols involving human subjects were approved by the Ethics Committees of the Affiliated Hospital of Guangdong Medical University, The First Affiliated Hospital of Harbin Medical University, the Central People’s Hospital of Zhanjiang, the First People’s Hospital of Pingdingshan, and Beijing Tongren Hospital affiliated with Capital Medical University and were executed in accordance with the Declaration of Helsinki. Informed consent documents were signed by participants at the time of human subject enrollment.

A total of 335 TLE patients and 325 healthy controls from the First Affiliated Hospital of Harbin Medical University, the Affiliated Hospital of Guangdong Medical University and Beijing Tongren Hospital affiliated with Capital Medical University were consecutively enrolled in cohort 1. To confirm the initial statistics observed in cohort 1, a validating cohort (cohort 2), including 161 TLE patients and 203 healthy controls from the First People’s Hospital of Pingdingshan and the Central People’s Hospital of Zhanjiang, was consecutively established. The human subjects in both cohorts 1 and 2 were Han Chinese, and the combined cohort (cohorts 1 + 2) was composed of 496 TLE patients and 528 healthy controls.

Gender, age, age at onset, and severity of disease (seizure frequency and drug response) of all participants were recorded via field investigation or telephone interviews. The inclusion criteria for TLE were based on typical temporal auras and temporal discharges at seizure onset observed by video-electroencephalograph, and subjects who failed to be genotyped were excluded from the study. According to the consensus about drug-resistant epilepsy proposed in 2010 by the Commission of the International League Against Epilepsy (Kwan et al., 2010), the responses to drug treatments were grouped as follows: drug-resistant patients are subjects with the absence of a significant change, an insufficient reduction in seizure frequency (<60%), or even an augmentation after 1 year treatment with a schedule of not less than two tolerated and properly collected anti-epileptic medicines; the remaining were considered drug-sensitive patients.

### DNA Preparation and Genotyping

First, 2 ml peripheral blood samples were collected from each human participant. DNA samples were extracted from blood samples using a Genomic DNA Extraction Kit (Tiangen Biotech, Beijing, China) and then used for genotyping the six *DTNBP1* SNPs (rs760665, rs1011313, rs2619528, rs2619522, rs909706, and rs2619538) via an ABI PRISM SNaPshot system (Applied Biosystems, Carlsbad, CA, United States). The primers used in a multiplex polymerase chain reaction (PCR) for the amplification of the six target fragments (338, 354, 274, 164, 278, and 183 nucleotides, respectively) were as follows: rs760665, 5′- cagccttggcctccaaacat-3′ (forward primer) and 5′-ttagccccagaaatgttattgatttga-3′ (reverse primer); rs1011313, 5′-ccagcatggagacgccaagt-3′ (forward primer) and 5′-tctgaagccttgaacccctcaa-3′ (reverse primer); rs2619528, 5′-tggttgcctgggatggagag-3′ (forward primer) and 5′-cgcactgaaacag tcttttcccatt-3′ (reverse primer); rs2619522, 5′-tgtttgggctctta tgtctacctttcc-3′ (forward primer) and 5′-gcccatttatctgttttccagtgc-3′ (reverse primer); rs909706, 5′-ggtgacaggctaatggcacaca-3′ (forward primer) and 5′-ttctggagtgggctctggactg-3′ (reverse primer); and rs2619538, 5′-ttggatgaggccagtgaggtaa-3′ (forward primer) and 5′-gaggtggtcccccagctacaag-3′ (reverse primer). The primers used for one-nucleotide extension in SNaPshot PCR were 5′-ttttttttttttttttttttttttttacaatagccttgctactatttaggggataaaca-3′ (rs760665), 5′-tttttttttttttcacaggctacagaatggatgttgc-3′ (rs10113- 13), 5′-tttggattggatgcacaaccatgtgaa-3′ (rs2619528), 5′-tttttttttttttt ttttttttttgcagaagcagtgagtgagagctgaca-3′ (rs2619522), 5′-tttttttttttt ttttgtcaagtcagtttccaaggggttctaac-3′ (rs909706), and 5′-tttttttttttttt ttttttttttgcaaagcttaataagacagagcagtttacatc-3′ (rs2619538).

The multiplex PCR mix was composed of 1x HotStarTaq buffer, 0.3 mM dNTP, 3.0 mM Mg^2+^, 1 μl of primer mix, 1 U of HotStarTaq polymerase, and 1 μl of the DNA template. The reaction mix was then run as follows: 1 cycle for 95°C/120 s; 11 cycles for 94°C/20 s, 65°C/40 s, and 72°C/90 s; 24 cycles for 94°C/20 s, 59°C/30 s, and 72°C/90 s; and 1 cycle for 72°C/120 s. Subsequently, the reaction products were purified using shrimp alkaline phosphatase and exonuclease I. The SNaPshot PCR mix contained 5 μl of SNaPshot Multiplex Kit, 1 μl of primer mix, 2 μl of ultrapure H_2_O, and 2 μl of the purified reaction products. The experimental program for a one-nucleotide extension was as follows: 1 cycle of 96°C/60 s; 28 cycles of 96°C/10 s, 55°C/5 s, and 60°C/30 s; and 1 cycle of 4°C/120 s. After further purification by shrimp alkaline phosphatase, the final products were analyzed using an ABI 3730xl DNA Analyzer and GeneMapper 4.1 (Applied Biosystems, Carlsbad, CA, United States).

### Dual-Luciferase Reporter Assay

In light of the *Homo sapiens* chromosome 6, GRCh37.p13 primary assembly, a 2 kb sequence upstream of the *DTNBP1* transcription start site harboring the T or A alleles at rs2619538 was cloned and individually ligated into pGL3 basic to create p*DTNBP1*-Promoter-Wildtype and p*DTNBP1*-Promoter-Mutant plasmids. In addition to the plasmids of the negative control (NC) and the positive control (PC), both of these plasmids were amplified in DH5α cells. Their positive clones were confirmed by further sequencing. Subsequently, HEK-293T cells were plated at 2 × 10^4^ cells per well in 24-well dishes, and 24 h later, the cells were individually cotransfected with four types of plasmids with the assistance of X-tremeGENE HP reagent (Roche, Basel, Switzerland). These four plasmids included 1 μg of pGL3 basic as the NC, 1 μg of pGL3 promoter as the PC, 1 μg of pSMG6-Promoter-Wildtype (Wild-type), and 1 μg of pSMG6-Promoter-Mutant (Mutant). Each cotransfection reaction was replicated four times, and the pRL plasmid served as an internal reference. After 24 h, the cotransfected cells were identified using a Dual-Luciferase^®^ Reporter Assay System (Promega, Madison, WI, United States), and both the firefly and Renilla luciferase activities were measured using a microplate luminometer (BioTek, VT, United States). The mutant impact of rs2619538 T > A was evaluated by dividing the averaged firefly/Renilla ratio of the mutant construct by the averaged firefly/Renilla ratio of the wild-type construct.

### Epilepsy Rat Model

Referring to our previous research (Tao et al., 2018), a total of 15 male Sprague Dawley (SD) rats from the Animal Center of Guangdong Medical University, Zhanjiang, China, were bred for adapting to the experimental environment at a temperature between 22 and 26°C and a humidity between 55 and 65%. The light-dark cycle was synchronous with a natural day-and-night cycle. After a 1 week adaptation period with free access to food and water, the SD rats (310 ± 32 g) were used in the following experiments: 10 rats were randomly selected to be administered pentetrazol (PTZ, 60 mg/kg body weight, i.p.; Sigma-Aldrich, St. Louis, MO, United States). The severity of epileptic seizures was classified in five levels according to the Racine scale: (1) twitching of facial muscle; (2) nodding of the head; (3) unilateral forelimb with lifting or clonus; (4) bilateral forelimb with clonus when standing; (5) falling when standing or twisting (Glen et al., 2014; Orozco et al., 2014). The administration of PTZ was repeated every 20 min during a 2 h observation period (10 mg/kg body weight, i.p.) until seizures increased to level 4 or the total dose of PTZ reached 90 mg/kg body weight. Finally, with the exception of one rat reaching level 4 for a duration of only 3 min, nine rats exhibited seizures that reached level 4 with a duration of 60 min and were immediately enrolled in the experimental group, followed by the administration of diazepam (10 mg/kg body weight, i.p.; Sigma-Aldrich, St. Louis, MO, United States) every 5 min until seizure cessation to reduce unexpected deaths before being sacrificed. The remaining five rats were classified as the control group. All animal experiments in this study were performed in accordance with the “Guide for the Care and Use of Laboratory Animals” (He et al., 2006), which was approved by the Animal Ethics Committee of Guangdong Medical University, Zhanjiang, China.

### Molecular Experiments

After a 2 h observation period, all rats in the experimental and control groups were sacrificed through decapitation under deep anesthesia (3% chloral hydrate, 10 ml/kg body weight, i.p.; Sigma-Aldrich, St. Louis, MO, United States). Then, the left and right hippocampi of nine experimental rats and four control rats were rapidly collected for use in real-time quantitative PCR (qPCR) and enzyme-linked immunosorbent assay (ELISA), respectively: (a) real-time qPCR: total RNA was extracted via an RNA extraction kit (QIAGEN Sciences, Germantown, United States), and reverse transcription was performed by using a First Strand cDNA Synthesis Kit (Thermo Fisher Scientific, Waltham, United States) according to the manufacturer’s instructions. Subsequently, the cDNA products were amplified using a Light-Cycler 480 sequence detector system (Roche Applied Science, Penzberg, Germany), and the specific primers used for real-time qPCR were as follows: *DTNBP1* forward primer, 5′-ttagcaggtatgaggatgcgt-3′, and reverse primer, 5′-ggtgcagcaaatggttctctac-3′. Relative expression levels were calculated by the 2^–ΔΔCT^ method. And (b) ELISA: *DTNBP1* concentrations in the hippocampi were measured using an ELISA kit (R&D Systems, Minneapolis, MN, United States) according to the manufacturer’s instructions. Absorbance was detected using an ELISA reader (Bio-Rad Laboratories, Hercules, CA, United States).

In addition to qPCR and ELISA, immunohistochemistry (IHC) was performed as follows: one experimental rat and one control rat under deep anesthesia were cardially perfused with physiological saline, followed by 4% paraformaldehyde, and their brains were then isolated and fixed in 4% paraformaldehyde at 4°C for 20 h. After dehydration and paraffin embedding, the specimens were cut into 4-μm-thick slices for subsequent histological staining. Paraffin-embedded slices were dewaxed with xylene and ethanol. Deparaffinized slices were rinsed using water, placed in a tissue slide, and heated in a microwave oven for antigen retrieval. Endogenous peroxidase was then blocked using 3% hydrogen peroxide. After three 5 min washes with phosphate buffer saline (PBS), the slices were blocked using 3% bovine serum albumin. Subsequently, the primary antibody (*DTNBP1* polyclonal antibody, ABclonal, Boston, MA, United States) was diluted in blocking solution and incubated overnight at 4°C. The next day, the slices were washed three times for 5 min each in PBS and then incubated with the secondary antibody, washed, developed with 3-3′ diaminobenzidine tetrachloride, and washed to stop the reaction until brown color appeared. The slices were counterstained with hematoxylin, dehydrated and mounted, and finally photographed for biological analysis.

### *DTNBP1* Expression in TLE Patients

To identify whether *DTNBP1* is abnormally expressed in human epilepsy patients, we used the online GSE datasets because of the lack of pathological brain samples of epilepsy patients in our laboratory. Unfortunately, after a series of searches in the online GSE datasets, we could not identify a single study that could be directly used. However, we found that GSE63808 was based on surgically acquired hippocampi from 129 TLE patients, and GSE29378 contained 32 autopsy samples of normal hippocampi. Moreover, both of these GSE datasets were acquired via the same technique, Illumina HumanHT-12 V3.0 expression BeadChip arrays (Johnson et al., 2015), and therefore, combining these two human transcriptome arrays makes it possible to study the expression of *DTNBP1* between epilepsy patients and normal controls. Hence, GSE63808 and GSE29378 were downloaded from the online GSE datasets. In total, 129 TLE patients from the GSE63808 dataset and 32 normal controls form the GSE29378 dataset were individually enrolled in the TLE and control groups, respectively, in the present study. After normalization and testing of multiple hypotheses to reduce the false-positive rate, the average signals of *DTNBP1* mRNA expression in the hippocampi of the TLE and control groups were compared for further analysis.

### Statistical Analyses

Variable data, reported as the mean ± SD, and attribute data were analyzed with a Student’s *t*-test and a chi-square test, respectively. Logistic regression was employed to correct for bias related to confounding factors, including age and gender, and q-values were used to adjust the false-positive results from multiple statistics by the Bonferroni correction. Statistical tests were performed using SPSS 19.0 (IBM, New York, NY, United States), and *p* ≤ 0.05 was considered statistically significant. In addition, haplotype construction and power analyses were carried out via Haploview 4.2 (Daly Lab, Cambridge, MA, United States) and Quanto 1.2 (University of Southern California, Los Angeles, CA, United States), respectively.

## Results

### General Characteristics of the Enrolled Cohorts

This study enrolled a total of 496 TLE patients and 528 healthy individuals. No significant differences in gender or age were observed between the TLE patients and the healthy controls in cohorts 1, 2, and 1 + 2 (all *p* > 0.05). Gender, age, duration of disease and severity of disease in cohorts 1 + 2 are listed in Table 1.

**TABLE 1:** General characteristics of enrolled cohorts.

	Cases	Controls	*p*-values
**Sex (male/female, n)**			
Cohort 1	170/165	185/140	0.112
Cohort 2	79/82	104/99	0.682
Cohorts 1 + 2	249/247	289/239	0.147
**Age (mean ± SD, years)**			
Cohort 1	31.2 ± 14.7	31.0 ± 9.7	0.862
Cohort 2	33.5 ± 14.9	32.7 ± 15.6	0.617
Cohorts 1 + 2	31.5 ± 14.8	31.7 ± 12.3	0.743
**Other characteristics in cohorts 1 + 2**			
Age at onset (mean ± SD, years)	20.8 ± 13.8	–	–
Severity of disease			
Seizure frequencies (mean ± SD, times/month)	6.7 ± 2.7	–	–
Drug response (sensitive/resistant patients, n)	250/246	–	–

In the present study, all enrolled patients and controls were successfully genotyped for the six *DTNBP1* SNPs (rs760665, rs1011313, rs2619528, rs2619522, rs909706, and rs2619538). The frequency distributions of these SNPs complied with the Hardy-Weinberg equilibrium. Power analyses indicated that this study had 12.9% power for rs760665, 99.7% power for rs1011313, 97.5% power for rs2619528, 97.5% power for rs2619522, 99.6% power for rs909706 and 87.2% power for rs2619538 for detecting a recessive inheritance model with a genetic effect of 1.8 at a significance level of 0.05 for two-sided type 1 error.

### Case-Control Analyses of the Six *DTNBP1* SNPs

In cohorts 1 and 2, the frequencies of the *TT* genotype at rs909706 T > C were consistently lower in the patients than in the controls (Table 2: *p* = 0.000 and 0.018, respectively). The same trend was observed after the Bonferroni correction for multiple comparisons in cohorts 1 + 2 (Table 3; OR = 1.770, *p* = 0.000 and *q* = 0.050), which confirmed the findings in cohorts 1 and 2. These results indicate that the *TT* genotype is associated with protection against TLE. In addition, no consistent differences were found for the remaining five SNPs (rs760665, rs1011313, rs2619528, rs2619522, and rs2619538) in cohorts 1, 2, and 1 + 2 (Tables 2, 3).

**TABLE 2:** Inheritance models of the *DTNBP1* SNPs in cohorts 1 and 2.

	Cohort 1				Cohort 2			
	Cases n (%)	Controls n (%)	ORs (95% CI)	*p*-values	Cases n (%)	Controls n (%)	ORs (95% CI)	*p*-values
**rs760665 T > A**								
*TT*/TA/AA	335 (100.0)/0.0)/0 (0.0)	323 (99.4)/2 (0.6)/0 (0.0)	–	0.113	161 (100.0)/0 (0.0)/0 (0.0)	202 (99.5)/1 (0.5)/0 (0.0)	–	1.000
*TT*/TA + AA	335 (100.0)/0 (0.0)	323 (99.4)/2 (0.6)	0.78 (0.57–1.06)	0.999	161 (100.0)/0 (0.0)	202 (99.5)/1 (0.5)	–	1.000
*TT* + TA/AA	335 (100.0)/0 (0.0)	325 (100.0)/0 (0.0)	–	0.999	161 (100.0)/0 (0.0)	203 (99.5)/0 (0.5)	–	–
**rs1011313 C > T**								
CC/CT/*TT*	221 (66.0)/98 (29.3)/16 (4.8)	182 (56.0)/131 (40.3)/12 (3.7)	0.75 (0.57–0.98)	0.034	105 (65.2)/48 (29.8)/8 (5.0)	114 (56.2)/81 (39.9)/8 (3.9)	0.78 (0.54–1.12)	0.172
CC/CT + *TT*	221 (66.0)/114 (34.0)	182 (56.0)/143 (4.0)	0.64 (0.47–0.88)	0.006	105 (65.2)/56 (34.8)	114 (56.2)/89 (43.8)	0.68 (0.44–1.04)	0.073
CC + CT/*TT*	319 (95.2)/16 (4.8)	313 (96.3)/12 (3.7)	0.81 (0.37–1.74)	0.585	153 (95.0)/8 (5.0)	195 (96.1)/8 (3.9)	0.79 (0.29–2.19)	0.656
**rs2619528 C > T**								
CC/CT/*TT*	275 (82.1)/58 (17.3)/2 (0.6)	266 (81.8)/55 (16.9)/4 (1.2)	0.95 (0.66–1.37)	0.772	131 (81.4)/29 (18.0)/1 (0.6)	169 (83.3)/32 (15.8)/2 (1.0)	1.11 (0.67–1.84)	0.697
CC/CT + *TT*	275 (82.1)/60 (17.9)	266 (81.8)/59 (18.2)	0.98 (0.66–1.46)	0.923	131 (81.4)/30 (18.6)	169 (83.3)/34 (16.7)	1.15 (0.67–1.98)	0.617
CC + CT/*TT*	333 (99.4)/2 (0.6)	321 (98.8)/4 (1.2)	2.10 (0.38–11.57)	0.395	160 (99.4)/1 (0.6)	201 (99.0)/2 (1.0)	1.54 (0.14–17.16)	0.727
**rs2619522 A > C**								
AA/AC/CC	277 (82.7)/56 (16.7)/2 (0.6)	266 (81.8)/55 (16.9)/4 (1.2)	1.09 (0.75–1.58)	0.641	132 (82.0)/28 (17.4)/1 (0.6)	169 (83.3)/32 (15.8)/2 (1.0)	0.94 (0.56–1.56)	0.807
AA/AC + CC	277 (82.7)/58 (17.3)	266 (81.8)/59 (18.2)	0.94 (0.63–1.41)	0.774	132 (82.0)/29 (18.0)	169 (83.3)/34 (16.7)	1.10 (0.64–1.90)	0.729
AA + AC/CC	333 (99.4)/2 (0.6)	321 (98.8)/4 (1.2)	2.10 (0.38–11.57)	0.395	160 (99.4)/1 (0.6)	201 (99.0)/2 (1.0)	1.54 (0.14–17.16)	0.727
**rs909706 T > C**								
*TT*/TC/CC	128 (38.2)/169 (50.4)/38 (11.3)	172 (52.9)/121 (37.2)/32 (9.8)	0.69 (0.55–0.87)	0.002	64 (39.8)/78 (48.4)/19 (11.8)	106 (52.2)/76 (37.4)/21 (10.3)	0.74 (0.54–1.00)	0.052
*TT*/TC + CC	128 (38.2)/207 (61.8)	172 (52.9)/153 (47.1)	1.83 (1.34–2.49)	0.000	64 (39.8)/97 (60.2)	106 (52.2)/97 (47.8)	1.66 (1.09–2.53)	0.018
*TT* + TC/CC	297 (88.7)/38 (11.3)	293 (90.2)/32 (9.8)	0.88 (0.53–1.45)	0.612	142 (88.2)/19 (11.8)	182 (89.7)/21 (10.3)	0.88 (0.45–1.69)	0.691
**rs2619538 T > A**								
*TT*/TA/AA	307 (91.6)/27 (8.1)/1 (0.3)	291 (89.5)/34 (10.5)/0 (0.0)	1.28 (0.75–21.17)	0.363	147 (91.3)/14 (8.7)/0 (0.0)	185 (91.1)/18 (8.9)/0 (0.0)	1.00 (0.48–2.08)	0.993
*TT*/TA + AA	307 (91.6)/28 (8.4)	291 (89.5)/34 (10.5)	–	–	147 (91.3)/14 (8.7)	185 (91.1)/18 (8.9)	–	–
*TT* + TA/AA	334 (99.7)/1 (0.3)	323 (100.0)/0 (0.0)	0.78 (0.46-1.33)	0.363	146 (100.0)/0 (0.0)	203 (100.0)/0 (0.0)	1.00 (0.48–2.10)	0.993

**p-values have been adjusted for sex and age.**

**TABLE 3:** Inheritance models of the SRR SNPs in cohorts 1 + 2.

	Cases n (%)	Controls n (%)	ORs (95% CI)	*p*-values	*q*-values
**rs760665 A > T**					
AA/AT/*TT*	496 (100.0)/0.0)/0 (0.0)	525 (99.4)/3 (0.6)/0 (0.0)	–	0.999	17.982
AA/AT + *TT*	496 (100.0)/0 (0.0)	525 (99.4)/3 (0.6)	–	0.999	17.982
AA + AT/*TT*	496 (100.0)/0 (0.0)	528 (100.0)/0 (0.0)	–	–	–
**rs1011313 C > T**					
CC/CT/*TT*	326 (65.7)/146 (29.4)/24 (4.8)	296 (56.1)/212 (40.2)/20 (3.8)	0.76 (0.61–0.94)	0.011	0.198
CC/CT + *TT*	326 (65.7)/170 (34.3)	296 (56.1)/232 (43.9)	0.66 (0.51–0.84)	0.001	0.018
CC + CT/*TT*	472 (95.2)/24 (4.8)	508 (96.2)/20 (3.8)	0.81 (0.44–1.49)	0.497	8.946
**rs2619528 C > T**					
CC/CT/*TT*	406 (82.1)/87 (17.3)/3 (0.6)	435 (82.4)/87 (16.5)/6 (1.1)	1.00 (0.74–1.35)	0.994	17.892
CC/CT + *TT*	406 (82.1)/90 (17.9)	435 (82.4)/93 (17.6)	1.04 (0.75–1.43)	0.833	14.994
CC + CT/*TT*	493 (99.4)/3 (0.6)	522 (98.9)/6 (1.1)	1.87 (0.47–7.54)	0.377	6.786
**rs2619522 A > C**					
AA/AC/CC	409 (82.5)/84 (16.9)/3 (0.6)	435 (82.4)/87 (16.5)/6 (1.1)	1.04 (0.77–1.40)	0.814	14.652
AA/AC + CC	409 (82.5)/87 (17.5)	435 (82.4)/93 (17.6)	0.99 (0.72–1.37)	0.973	17.514
AA + AC/CC	493 (99.4)/3 (0.6)	522 (98.9)/6 (1.1)	1.87 (0.47–7.54)	0.377	6.786
**rs909706 T > C**					
*TT*/TC/CC	192 (38.7)/247 (49.8)/57 (11.5)	278 (52.7)/197 (37.3)/53 (10.0)	0.71 (0.59–0.85)	0.000	0.005
*TT*/TC + CC	192 (38.7)/304 (61.3)	278 (52.7)/250 (47.3)	1.77 (1.38–2.27)	0.000	0.000
*TT* + TC/CC	439 (88.5)/57 (11.5)	475 (90.0)/53 (10.0)	0.88 (0.59–1.30)	0.517	9.306
**rs2619538 T > A**					
*TT*/TA/AA	454 (91.5)/41 (8.3)/1 (0.2)	476 (90.2)/52 (9.8)/0 (0.0)	1.14 (0.75–1.74)	0.539	9.702
*TT*/TA + AA	454 (91.5)/42 (8.5)	476 (90.2)/52 (9.8)	0.85 (0.56–1.31)	0.464	8.352
*TT* + TA/AA	495 (99.8)/1 (0.2)	528 (100.0)/0 (0.0)	–	1.000	18.000

**ORs and p-values have been adjusted for sex and age; considering the number of statistical comparisons reached 18 times, Bonferroni correction was employed to adjust the false positive rate, and the q-value of each comparison was equal to 18 times its p-value.**

Through the use of Haploview 4.2, a haplotype block (rs1011313-rs2619528-rs2619522-rs909706) approximately 27 kb in length was constructed based on all six SNPs including four haplotypes (*CCAT, CCAC, TCAT*, *and CTCC*). Their frequencies were then compared between the patients and the controls in cohorts 1 + 2. As displayed in Table 4, the patient ratios of the CCAC and TCAT haplotypes were lower than the control ratio (16.3 vs. 19.2%, *p* = 0.000, and 13.7 vs. 23.8%, *p* = 0.006), which was further confirmed by the Bonferroni correction for multiple comparisons (q = 0.000 and 0.025). Thus, the CCAC and TCAT haplotypes could be genetically protective markers against susceptibility to TLE.

**TABLE 4:** Haplotypes of the *DTNBP1* SNPs in cohorts 1 + 2.

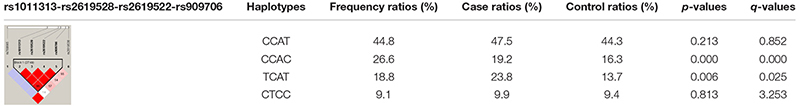

**q-values were calculated with Bonferroni correction; considering the number of statistical comparisons reached 4 times, Bonferroni correction was employed to adjust the false positive rate, and the q-value of each comparison was equal to 4 times its p-value.**

### Genetic Significance of the TT Genotype at rs909706

**FIGURE 1:**
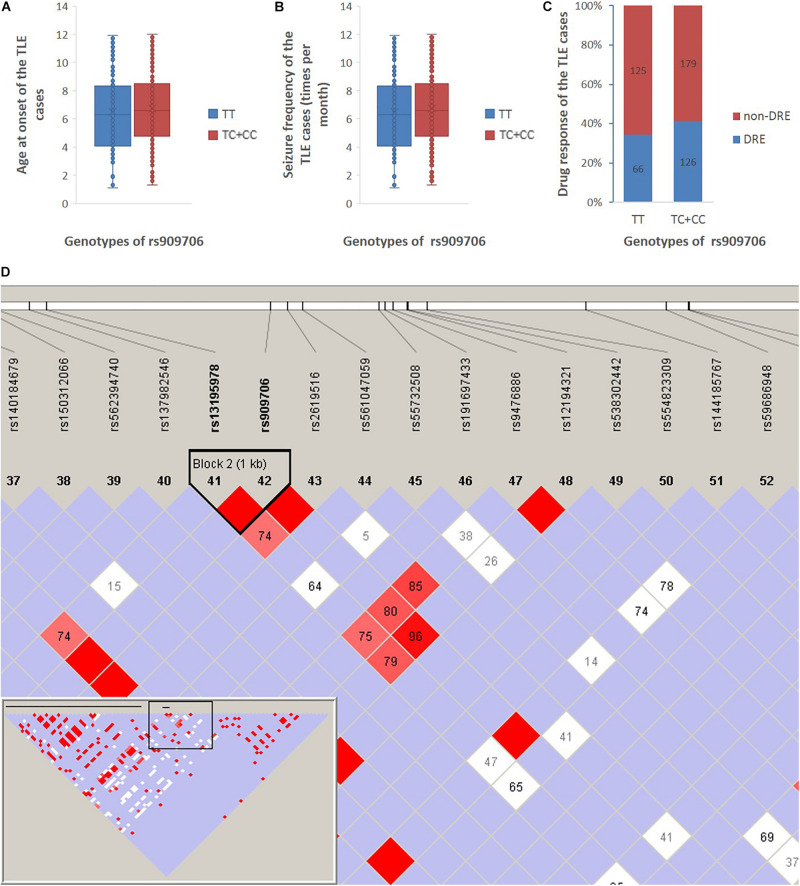
Age at onset, seizure frequency, and drug response between the *TT* genotype and the *TC* + *CC* genotypes in all TLE patients in cohort 1 + 2. **(A)** The age at onset of the TLE patients was 22 ± 16 and 20 ± 13 years among those with the *TT* genotype and the *TC* + *CC* genotypes, respectively. **(B)** Seizure frequency of patients with the *TT* genotype and the *TC* + *CC* genotypes was 6.5 ± 2.7 and 6.7 ± 2.6 per month, respectively. **(C)** Drug-resistant incidence of patients with the *TT* genotype and the *TC* + *CC* genotypes was 34.4 and 41.1%, respectively. **(D)** According to the 1,000 Genomes Project, SNPs are downloaded between 10,000 nucleotides downstream and 10,000 nucleotides upstream of the rs909706 locus (Chromosome 6, NC_000006.11 (15650871..15670871), and according to the analysis using Haploview 4.2, only rs13195978 was observed to be in LD with rs909706 (*D*’ = 1.0).

Given the findings of case-control analysis, we further investigated the genetic impact of the wild-type homozygote at rs909706 T > C on age at onset, seizure frequency, and drug response in cohorts 1 + 2. As shown in Figure 1, no significant differences in age at onset, seizure frequency or drug response in the TLE patients were found between carriers with the *TT* genotype and those with the *TT* + *TG* genotypes (*p* = 0.221, 0.349, and 0.132), and similar results occurred after adjusting for gender and age (*p* = 0.202, 0.254, and 0.130).

According to the assembly of the human genome GRCh38.p7, rs909706 is located in the first intron of the DTNBP1 gene. However, introns are non-coding sequences, and variations within them rarely modulate gene function. Thus, we decided to investigate whether other functional SNPs are in linkage disequilibrium (LD) with variations at rs909706. As shown in Figure 1, rs13195978 was found to be in LD with rs909706, but rs13195978 is still non-functional due to its locus in the second intron of the *DTNBP1* gene. Therefore, there are no clues to explain the protective role of the *TT* genotype at rs909706 against susceptibility to TLE.

### Impact of Homozygous Mutation of rs2619538 T > A on *DTNBP1* Transcription

**FIGURE 2:**
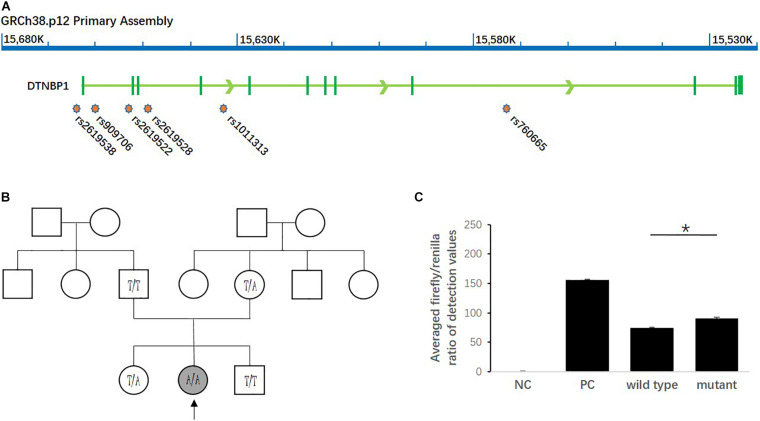
Impact of the homozygous mutation of rs2619538 T > A on DTNBP1 transcription. **(A)** Relative loci of rs2619538 T > A and other SNPs genotyped in this study are shown in the DNTBP1 gene region. Only rs2619538 T > A is potentially functional because of its location in the predicted promoter of the DNTBP1 gene, and yet the remaining SNPs are located within several introns of the DTNBP1 gene. **(B)** The TLE patient carrying the homozygous mutation of rs2619538 T > A was a 33-years-old male (age at onset, 32 years). After observation of the treatment for 1 year, seizure frequencies decreased from three times per month to one time per half year. A genealogical chart of his family was also constructed, and his parents consented to genotyping of the same site at rs2619538 T > A; a *novo* mutant allele probably coming from his father was observed. **(C)** The effects of rs2619538 T > A mutant on the activity of the DTNBP1 gene promoter. Compared with the averaged firefly/renilla ratios of the pGL3 basic negative control (NC), the ratios of the pGL3 promoter positive control (PC), pDTNBP1-Promoter-Wildtype (Wildtype) and pDTNBP1-Promoter-Mutant (Mutant) for rs2619538 T > A were 155.74 ± 1.43, 74.07 ± 0.99, and 90.48 ± 2.09, respectively. *p < 0.05.

As shown in Figure 2, among all the SNPs genotyped in the study, rs2619538 T > A is the only SNP located in the predicted promoter region of the *DTNBP1* gene; thus, its variation might affect *DTNBP1* transcription. Notably, homozygous mutation of rs2619538 T > A was rarely observed in the patient group (1/325) but not in the control group (0/335), suggesting that rs2619538 T > A might be a functional site involved in modulation of *DTNBP1* transcription. Given these clues, we further constructed a genealogical chart of the mutant patient and genotyped the same site of his parents and brothers/sisters, and we identified a *novo* mutant allele in his father. Hence, we precluded its potential impact on the activity of the *DTNBP1* gene promoter.

In support of the mutant impact of rs2619538 T > A on the *DTNBP1* gene promoter, we synthesized reporter gene constructs containing *T* (wild-type) or *A* (mutant) alleles in the context of the full-length promoter of *DTNBP1* (2 kb sequence upstream of the transcription start site). The dual-luciferase assay revealed that both wild-type and mutant constructs have biological activities in the full-length promoter of *DTNBP1*, and the latter increased by approximately 22% in comparison with the former (Figure 2). This finding indicates that the homozygous mutation at rs2619538 T > A could upregulate *DTNBP1* transcription.

### Expression Patterns of *DTNBP1* in an Epilepsy Rat Model and in TLE Patients

**FIGURE 3:**
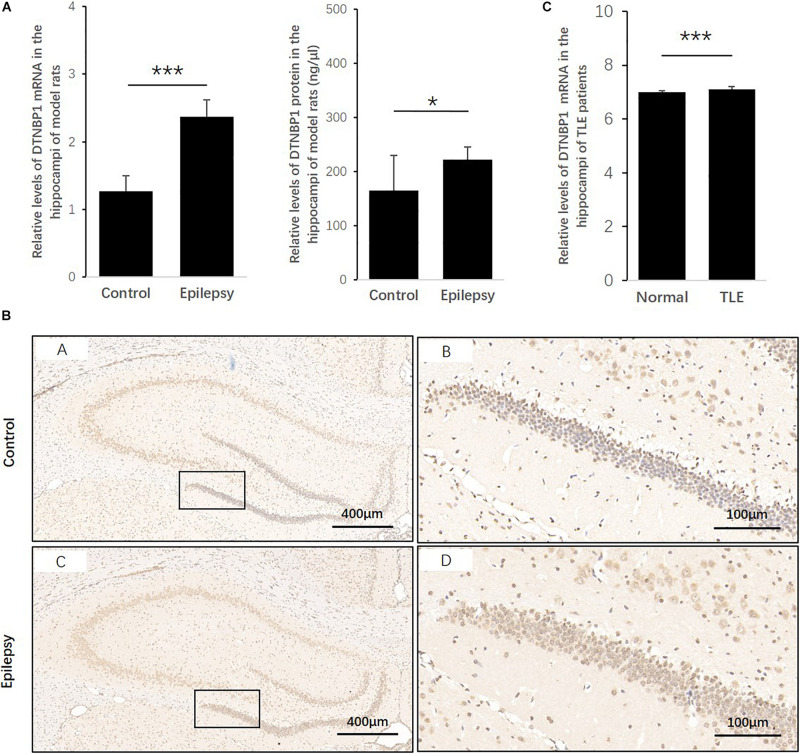
Expression patterns of *DTNBP1* in an epilepsy rat model and TLE patients. **(A)** According to qPCR, the relative levels of *DTNBP1* mRNA in the hippocampi of normal rats and epilepsy model rats were 1.27 ± 0.23 and 2.37 ± 0.25, respectively. **(B)** According to ELISA, the concentrations of *DTNBP1* protein in the hippocampi of normal rats and epilepsy model rats were 164.60 ± 64.93 and 222.42 ± 23.20 ng/μl, respectively. **(C)** A total of 129 TLE patients from the GSE63808 dataset and 32 normal controls from the GSE29378 dataset were enrolled in the TLE and control groups. After normalization and multiple hypothesis testing to reduce the false-positive rate, the average *DTNBP1* mRNA expression in the hippocampi of the TLE and control groups was 7.11 ± 0.09 and 6.99 ± 0.07, respectively. **(D)** IHC staining is shown individually for global and local regions of the hippocampi. Hematoxylin staining was used to mark the cell nuclei (blue), and immunostaining was used to show *DTNBP1* expression (brown). *p < 0.05, ***p < 0.001.

To determine whether *DTNBP1* expression is abnormal in epileptic activity, we established a rat epilepsy model according to a PTZ-induced protocol. *DTNBP1* mRNA and protein expression was detected in the hippocampi by qPCR and ELISA. Compared with control rats, the epilepsy model showed an approximately 87% increase (*p* = 0.000) in the expression of *DTNBP1* mRNA, similar to the increased rate of *DTNBP1* protein (approximately 35%, *p* = 0.044). In addition, IHC confirmed the abnormal expression of *DTNBP1* in the epilepsy model, indicating that hyperfunction of *DTNBP1* is involved in epileptic seizures (Figure 3).

To further identify whether *DTNBP1* is abnormally expressed in human epilepsy patients, we used online GSE datasets of pathological brain samples from TLE patients and normal controls that were acquired by the same technique, Illumina HumanHT-12 V3.0 expression BeadChip arrays. Despite the small amplitude increase of 1.74%, the level of *DTNBP1* expression in the hippocampi of the TLE patients was significantly higher than that of the normal controls (*p* = 9.316 × 10^–9^), suggesting that homozygous mutation at rs2619538 T > A might be involved in epileptic activity by upregulating *DTNBP1* expression.

## Discussion

This study first observed that the frequencies of the *TT* genotype at rs909706 T > C and the *CCAC* and *TCAT* haplotypes (rs1011313-rs2619528-rs2619522-rs909706) were significantly lower in TLE patients than in controls. A previous study reported that the concentration of hippocampal glutamate was significantly affected by variations in rs909706 T > C (Wirth et al., 2012), whereas in our analyses, the intronic *TT* genotype was not related to age at onset or severity of disease and was not in LD with any functional variations nearby. Hence, these findings are considered to indicate protective markers against genetic susceptibility to TLE.

Meanwhile, the homozygous mutation of rs2619538 T > A was rarely observed in TLE patients, and the *novo* mutant allele came from the father. The dual-luciferase assay revealed that *DTNBP2* promoter activity in patients with the mutant allele increased by approximately 22% compared with patients with the wild-type allele. Together with the trend of increased *DTNBP1* expression in epilepsy patients and animal models in the present study, we hypothesized that the homozygous mutation of rs2619538 T > A functions by promoting DTNBP1 expression and subsequent processes in epilepsy pathologies.

According to previous research, the homozygous mutation at rs2619538 T > A is associated with reduced volumes of both gray and white matter in healthy children as young as 10–12 years old (Tognin et al., 2011). Structural volume deficits were observed in cortical regions, the subiculum and dentate gyrus, and the striatum of *DTNBP1* mutant mice (Wirth et al., 2012). These findings indicate a key role of *DTNBP1* in brain development, and abnormalities of brain development often lead to cortical dysplasia and subsequent early conset seizures (Palmini and Holthausen, 2013; Liu et al., 2015; Shaker et al., 2016; Maynard et al., 2017). On the other hand, *DTNBP1* inhibits the release of glutamate (Chen et al., 2008; Saggu et al., 2013), which is crucial for AMPAR-mediated synaptic transmission and plasticity and NMDAR-dependent synaptic potentiation in the hippocampus (Glen et al., 2014; Orozco et al., 2014). In addition, *DTNBP1* null gene mutation was observed to influence the developmental switch between GluN2B and GluN2A in the mouse cortex and hippocampus (Sinclair et al., 2016). These findings further indicate that *DTNBP1* should be a critical modulator in excitatory signal transduction of glutamate, as well as brain development. Considering that an epilepsy model induced by PTZ is usually used to observe changes of excitatory/inhibitory regulatory molecules during the period of acute convulsions, this study successfully used the model to confirm increased DTNBP1 expression in the PTZ model, which indicates that the model can be used for disclosing potential mechanisms of DTNBP1 in future experiments.

Notably, *DTNBP1* facilitates neurite outgrowth by promoting the transcriptional activity of p53 (Ma et al., 2011), and nucleocytoplasmic shuttling of *DTNBP1* regulates synapsin I expression (Fei et al., 2010). However, neither overexpression (Dys1A-Tg) nor underexpression (Sandy) of *DTNBP1* cause epileptic seizures in mice (Shintani et al., 2014). Because these prior studies focused on schizophrenia, more attention was paid to the effects of low-level *DTNBP1* expression. In contrast, the expression of *DTNBP1* was demonstrated to be increased in TLE patients and an animal model in this study; thus, behavioral observations and electrophysiological results of epileptic seizures in Dys1A-Tg mice and the related mechanisms should be further evaluated in the future.

Several limitations of the present study should be noted. First, *DTNBP1* encodes dysbindin-1, which is composed of three spliceosomes, but only dysbindin-1B, not dysbindin-1A or dysbindin-1C, displays a tendency for toxic aggregation. In postmortem brains, dysbindin-1B not only aggregates with itself but also aggregates with proteins that interact with it (Xu et al., 2015; Zhu et al., 2015; Yang et al., 2016). Another study observed that increased expression of dysbindin-1A resulted in a selective deficit in NMDA receptor signaling in the hippocampus (Jeans et al., 2011). Additionally, dysbindin-1C is required for the survival of hilar mossy cells and the maturation of adult newborn neurons in the dentate gyrus (Wang et al., 2014), and dysbindin-1C deficiency could result in impaired autophagy (Yuan et al., 2015). Therefore, the expression patterns of the three spliceosomes most likely interfere with the function of *DTNBP1* and influence genetic susceptibility to TLE. Second, when we consider 1,000 genomes, the minor allele frequencies of rs2619538 T > A are 0.027 among those of east Asian ancestry, 0.645 among those of African ancestry, 0.549 among those of European ancestry and 0.42 among those of American ancestry, which indicates that the mutant effects of rs2619538 T > A in the Han Chinese population included in the present study probably represent a mechanism that is distinct from those of other ancestries. Thus, one should be cautious in generalizing our findings to other races. Third, in addition to SNPs at rs2619538 T > A, DNA methylation of the *DTNBP1* promoter also plays a key role in the expression of *DTNBP1* and related pathological activities (Wockner et al., 2014; Abdolmaleky et al., 2015); thus, DNA methylation is another potential factor that could interfere with the genetic effects of rs2619538 T > A.

## Conclusion

This study first demonstrated the association of *DTNBP1* with TLE from a genetic perspective. In particular, the homozygous mutation rs2619538 T > A was observed in TLE patients but not in healthy controls. The increased activities of the *DTNBP1* promoter with the A allele in dual-luciferase assays and increased *DTNBP1* expression in epilepsy patients and animal models suggest that the mutation likely functions by promoting transcription of the *DTNBP1* gene and facilitating subsequent processes in epilepsy pathologies. Hence, the role of *DTNBP1* in TLE deserves further exploration in the future.

## Data Availability Statement

The raw data supporting the conclusions of this article will be made available by the authors, without undue reservation.

## Ethics Statement

All experimental protocols involving human subjects were approved by the Ethics Committees of the Affiliated Hospital of Guangdong Medical University, The First Affiliated Hospital of Harbin Medical University, the Central People’s Hospital of Zhanjiang, the First People’s Hospital of Pingdingshan, and Beijing Tongren Hospital affiliated with Capital Medical University. The patients/participants provided their written informed consent to participate in this study. The animal study was reviewed and approved by the Ethics Committees of Guangdong Medical University. Written informed consent was obtained from the owners for the participation of their animals in this study. Written informed consent was obtained from the individual(s) for the publication of any potentially identifiable images or data included in this article.

## Author Contributions

HT and XZ undertook data analyses and wrote the manuscript. JC, HZ, LH, and YCa carried out biological experiments. JF, ZL, and YCh carried out specimen collection. CS carried out epilepsy model. BZ, WZ, and KL conceptualized the hypothesis and designed the study. All authors contributed to the article and approved the submitted version.

## Conflict of Interest

The authors declare that the research was conducted in the absence of any commercial or financial relationships that could be construed as a potential conflict of interest.
